# Diabetes decreases patency of tunneled catheters in hemodialysis patients after first effective thrombolysis with urokinase

**DOI:** 10.1080/0886022X.2018.1487856

**Published:** 2018-07-16

**Authors:** Dominika Wójtowicz, Dominika Cholewa, Anna M. Faba, Beata Domańska, Joanna Kokoszka, Konrad Kopacz, Rafał Ficek, Tomasz Irzyniec, Sylwia E. Rotkegel, Jerzy Chudek

**Affiliations:** a Pathophysiology Unit, Department of Pathophysiology, School of Medicine in Katowice, Medical University of Silesia in Katowice, Katowice, Poland;; b Dialysis Center in Katowice "NEFROMED", Centrum Dializa Sosnowiec, Katowice, Poland;; c Department of Nephrology, Transplantation and Internal Medicine, School of Medicine in Katowice, Medical University of Silesia in Katowice, Katowice, Poland;; d Department of Nephrology with Dialysis Unit, MSWiA Hospital, Katowice, Poland;; e Department of Health Promotion and Community Nursing, School of Health Sciences, Medical University of Silesia in Katowice, Katowice, Poland;; f Department of Internal Medicine and Oncological Chemotherapy, School of Medicine in Katowice, Medical University of Silesia in Katowice, Katowice, Poland

**Keywords:** Permanent catheter, thrombolysis, urokinase, recurrent thrombosis

## Abstract

**Introduction:** Fibrinolysis is one of the methods extending the use of vascular access in patients with tunneled venous catheters thrombosis. The aim of this study was to assess one-year maintenance of tunneled catheters patency after first effective thrombolysis with urokinase and identify its predictors.

**Methods:** Retrospective analysis included 85 patients (age 69 ± 13 years) with permanent venous catheter thrombosis treated with urokinase at one center in the period 2010–2016. Urokinase was used (depending on weight) at a dose of 10,000 or 20,000 IU in an 8 h infusion to each catheter line. Assessment of one-year efficacy of fibrinolysis included the time between fibrinolysis and following thrombosis of the same catheter in patients that have previously obtained at least partial blood flow. The analysis included medication, comorbidities, catheter patency time and INR value during first thrombosis episode.

**Results:** There were 62.4% patients with type-2 diabetes and 11.8% with neoplasm. The thrombolysis procedure was effective in 73 patients (85.9%). An analysis of the one-year efficacy of thrombolysis procedure included 73 patients. Among them, 23 experienced next episode of catheter-related thrombosis within a year postprocedure. Diabetes increased the risk for recurrent thrombosis [HR =3.19 (1.09-9.41); *p* = .03].

**Conclusions:** Patients with diabetes are at higher risk of recurrent catheter-related thrombosis and therefore may require more aggressive anticoagulation therapy for its prevention.

## Introduction

Tunneled catheters are commonly used as permanent vascular access in hemodialysis patients for whom arteriovenous fistula formation is not recommended or several attempts to create a workable arteriovenous fistula have failed. The most common complication associated with their use is catheter-related thrombosis [1], which, together with infection, are serious therapeutic problems in this group of patients. Catheter-related thrombosis leads to the catheter malfunction, resulting in ineffective hemodialysis. Subsequent complications of catheter-related thrombosis include concomitant infection and pulmonary embolism [2]. Most episodes of thrombosis are associated with an activation of the external clotting pathway rather than coagulopathy [3].

According to Little et al., one-year tunneled catheter survival in hemodialysis is estimated at 47.5% [1]. During two and a half years observation 34% of patients with implanted tunneled catheters developed catheter malfunction or thrombosis [1], and at least some of them might benefit from thrombolytic procedures that extend the use of vascular access and prevent vein exhaustion [4].

We can distinguish primary and secondary management of catheter-related thrombosis. The primary procedures include all efforts to restore the catheter’s performance, which may be undertaken immediately when the occlusion is diagnosed. They include forceful flush, fibrinolytic enzyme instillation, infusion and mechanical therapy. If they are not effective, removal of the catheter or covering it fiber sheath, are considered as the secondary procedures [3].

Several thrombolytic agents are approved in the thrombolytic therapy, but the most commonly used are recombinant plasminogen activator (rtPa) – alteplase, and much cheaper urokinase. They catalyze the conversion of plasminogen into plasmin that initiates fibrinolysis of a clot, which can begin to form even 24 h after catheter implantation. Thrombolytics are noninvasive, confer no additional trauma to the patient, have a higher level of safety than catheter replacement and are cost-effective [5].

In the studies comparing both drugs, alteplase was slightly more effective (80–95%) and caused fewer allergic reactions (0.02% [6]); however, its superiority was most significant in cases when the vascular access was completely blocked [7]. Regardless of that, urokinase is still recommended by K/DOQI in 4 h infusion [8] and most frequently used a thrombolytic agent in the Europe [4]. It is cheaper and easier to store than alteplase [9]. Its efficacy in the preserving of vascular access was shown in multiple studies (81% [4] and 97% [9]).

The study by Pollo et al. showed that the choice of drug used for thrombolysis does not affect long-term catheter survival – no difference in patency during 10 subsequent hemodialysis sessions (93% vs. 85.7% *p* = .23 [10]). These data are in line with the previous study by Zacharias et al. [7].

Recurrent thrombosis nullifies the therapeutic effect of thrombolysis in some patients. However, the predisposing factors for recurrent thrombosis are hardly known. It seems that not all known factors increasing blood clotting (e.g., cancers, some autoimmune diseases, obesity, thrombophilia, respiratory and heart failure – NYHA class III and IV) are important for maintaining patency of catheter. Moreover, especially in a part of patients with recurrent thrombosis undiagnosed thrombophilia can be anticipated [11].

The aim of this study was to assess one-year maintenance of tunneled catheters patency after first effective thrombolysis with urokinase and identify its predictors.

## Methods

The study was a retrospective analysis of medical records of all thrombolytic treatments with urokinase (*N* = 85) performed in the Silesian Dialysis Centre in the years 2010–2016 and follow-up period based on hemodialysis protocols of the patients in 12 local hemodialysis units. As a retrospective analysis the study did not meet the criteria of the medical experiment, therefore did not require conscious consent and agreement from the bioethics committee.

Thrombolytic procedures were undertaken in patients with catheter dysfunction for at least two consequent HD sessions (with blood flow <180 mL/min) without indications for urgent hemodialysis (massive fluid overload, uncontrolled hypertension, resistant for pharmacotherapy hyperkalaemia >6.2 mmol/l), sign and symptoms of catheter-related bloodstream infection and contraindications for the use of urokinase included in Summary of Product Characteristics. Patients with complete catheter obstruction without any improvement after forced flashes, repositioning maneuvers and heparin instillation for one day were not qualified to the procedure.

Data concerning catheter dysfunction, its location, urokinase doses, blood flow setting after the thrombolytic procedure (for the analysis of catheter patency), medication before the treatment, comorbidities, and prior catheter dysfunctions were retrieved from medical records.

In all participating centers, low molecular weight heparins (LMWH) as an anticoagulant during HD session and heparin locks (5000 IU/ml) were used.

### Thrombolytic procedure

The thrombolytic procedure was performed in inpatients of the Department of Nephrology with 8 h infusion (dual syringe pomp) of urokinase (Medac Gesellschaft für Klinische, Wedel, Germany) dissolved in 0.9% sodium chloride, to each line of the tunneled catheter. The dose depended on patients’ body mass: 10,000 U in patients with body mass below 80 kg and 20,000 U in patients with mass over 80 kg. All patients before the procedure had controlled total blood count, activated partial thromboplastin time (aPTT), prothrombin time (PT), serum electrolytes and creatinine. Fibrinogen degradation products were not assessed. The effectiveness of the procedure was controlled during HD session performed immediately postprocedure.

### Data analysis

The early efficacy of thrombolytic procedure was assessed during the subsequent hemodialysis performed immediately after the urokinase infusion. The procedure was assessed as effective if sustained post-thrombolytic blood flow during the hemodialysis was at least 180 mL/min or partially effective if blood flow was slightly below 180 mL/min, but the patency was restored. The use 180 mL/min as the cut-point was based on our experience with hemodialysis procedures utilizing low-flux dialyzers that allow to obtain kt/V > 1.2 and URR >65%. The lack of patency postprocedure was followed by replacement of the catheter.

The late efficacy of thrombolytic procedure was analyzed only in patients with fully or partially effective initial procedure (patients necessitating replacement of the catheter were excluded). The end-point in this analysis was time to the first episode of the catheter malfunction due to catheter sheeting or thrombus (replacement or thrombolysis). The replacement of the catheter due to catheter-related infection ended the observation without reaching the end-point. The majority of patients with catheter malfunction were referred again to the Silesian Dialysis Centre.

### Statistical analysis

Statistical analyses were performed using STATISTICA 10.0 PL (StatSoft, Tulsa, Oklahoma). The results are presented as mean values ± standard deviation. Distribution of variables was evaluated by the Shapiro–Wilk test. Homogeneity of variances was assessed by the Levene test. Quantitative variables were compared with the parametric Student t-test. Logistic regression analysis was used for the selection of predictors of an early efficacy of thrombolysis. Ordinal and nominal data were compared with χ^2^ or Fisher exact test. Tunneled catheter survival curves were obtained by the Kaplan–Meier method. Equality of survival profiles between patients with and without potential predictors was examined by the log-rank test. In order to assess risk factors of death univariable Cox proportional hazard analysis was performed.

In all tests the *p* values <.05 were considered as statistically significant.

## Results

### Study group characteristics

Among the study group, there was 62.4% of patients with type-2 diabetes, and 11.8% was suffering from neoplasm. It was next episode of catheter-related thrombosis for 40 patients (47.1%). Thirty-four of patients (40%) were receiving antiplatelet drugs, 18 (21.2%) vitamin K antagonists and seven (8.2%) low molecular weight heparin (LMWH) between dialysis sessions. The location of permanent catheters is shown in Table 1.

**Table 1.** Patients’ characteristics [*N* = 85].

Age [years]	69 ± 13
Sex [men/women]	32/53
Previous catheter-related thrombosis [n/%]	40/47.1
	[*n*/%]
Concomitant diseases	
Hypertension	46/54.1
Ischaemic heart disease	49/57.6
Past myocardial infarction	17/20
Heart failure	11/12.9
Atrial fibrillation	20/23.5
Past stroke	9/10.6
Type 2 diabetes	53/62.4
Neoplasm	10/11.8
Anticoagulant /antiplatelet medication	
Aspirin	33/38.8
Clopidogrel/ticlopidin	3/3.5
Vitamin K antagonists	18/21.2
Low molecular weight heparin	7/8.2
Catheter localization	
Right internal jugular vein	56/65.9
Left internal jugular vein	12/14.1
Right femoral vein	12/14.1
Left femoral vein	4/4.7
Inferior vena cava	1/1.2

### Early efficacy of thrombolysis

The thrombolysis procedure was effective in 70 patients and partially effective in 3 (in total in 73 patients – 85.9%), while ineffective in 12 patients. Comparing both doses of urokinase used for thrombolysis, 2 × 20,000 U with 2 × 10,000 U, we did not observe expected higher efficacy for higher doses (13 effective +2 partially effective procedures/18 – 83.3% vs 57 effective +1 partially effective procedures/66 – 86.6%).

A search for factors limiting the early efficacy of thrombolysis failed to discriminate significant predictors (Table 2).

**Table 2.** Predictors of an early efficacy of thrombolysis with urokinase in patients with catheter-related thrombosis.

		Effective thrombolysis	Relative risk (95% CI)	*p*
Diabetes	Yes [*N* = 53]	46 (84.4%]	0.93 (0.56–1.54)	.77
	No [*N* = 32]	27 (88.5%)	Ref	
Neoplasm	Yes [*N* = 10]	8 (80%)	0.66 (0.16–2.73)	.56
	No [*N* = 75]	65 (86.7%)	ref	
Atrial fibrillation	Yes [*N* = 20]	19 (95%)	3.12 (0.46–21.2)	.24
	No [*N* = 65]	54 (83.1%)	ref	
Previous catheter-related thrombosis	Yes [*N* = 40]	33 (82.5%)	0.77 (0.45–1.33)	.35
	No [*N* = 45]	40 (88.9%)	ref	
Coumarines (vitamin K antagonists)	Yes [*N* = 18]	15 (83.3%)	0.82 (0.28–2.42)	.72
	No [*N* = 67]	58 (86.6%)	ref	
Aspirin	Yes [*N* = 33]	29 (87.9%)	1.19 (0.51–2.78)	.68
	No [*N* = 52]	44 (84.6%)	ref	
Any antiplatelet drug	Yes [*N* = 34]	30 (88.2%)	1.23 (0.53–2.87)	.63
	No [*N* = 51]	43 (84.3%)	ref	
Low molecular weight heparin	Yes [*N* = 7]	7 (100%)	2.64 (0.16–43.4)	.50
	No [*N* = 66]	66 (84.6%)	ref	

### Long-term efficacy of initially effective procedure

An analysis of the one-year efficacy of thrombolysis procedure included 73 patients with at least partially effective procedure. Among them, 23 experienced next episode of catheter-related thrombosis within a year postprocedure. The one-year postprocedure patency of the tunneled catheters was estimated at 68%. The majority (*N* = 17) of catheter-related thrombosis have occurred within two months.

**Figure 1.**
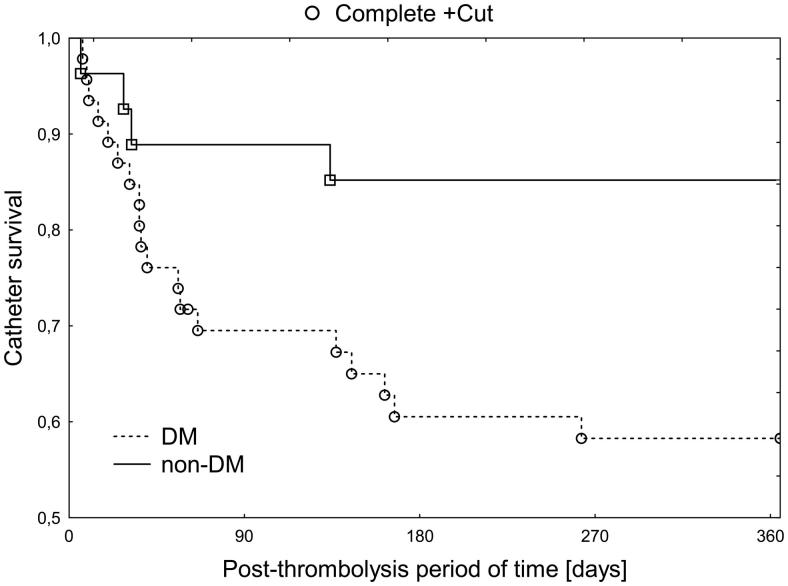
The effect of the occurrence of type-2 diabetes on catheter survival after effective thrombolysis procedure (*F* = 2.24, *p* = .02). Dotted line: patients with diabetes; continuous line: nondiabetic patients.

There was a strong effect of diabetes occurrence on one-year catheter survival due to catheter-related thrombosis [HR = .31 (0.11–0.92); *p* = .03] – (Table 3, Figure 1).

**Table 3.** Predictors of catheter survival (univariate models).

	HR (95% CI)	*p*
Diabetes	0.31 (0.11–0.92)	.03
Neoplasm	1.35 (0.32–5.88)	.69
Atrial fibrillation	0.88 (0.36–2.17)	.79
Previous catheter-related thrombosis	0.69 (0.30–1.56)	.37

## Discussion

In our one center study, we have analyzed the efficacy of urokinase in catheter-related thrombosis and then one-year maintenance of tunneled catheters patency when the procedure was successful. The obtained efficacy of thrombolysis with urokinase was 85.5% and is comparable to that obtained by other researchers [9]. A greater efficacy (97%) was described by Shavit et al. for a small subset of patients (*n* = 33), using a higher dose of urokinase (125,000 U) within 90 min [9].

The thrombolysis procedures with urokinase were safe. We did not observe any adverse event, including gastrointestinal bleeding, previously described by Shavit et al. [9]. Furthermore, we failed to find prognostic factors of early efficacy of the procedure, perhaps due to an insufficient sample size of the study group.

The one-year maintenance of tunneled catheters patency after first effective thrombolysis with urokinase was estimated at 68%, assuming a blood flow of less than 180 mL/min as insufficient, requiring physician intervention. Relatively high one-year patency rate might be a consequence of the use of LMWH between dialysis sessions, as well as low cutoff value for blood flow during HD sessions (<180 mL/min) demanding for next intervention.

It has to be stressed that there is no clear definition of catheter loss in the literature, as it should take into account not only the blood flow through the catheter, but also other factors affecting the dialysis efficacy [4,12]. This partially explains variations in published data. Most commonly expected post-thrombolytic blood flow for low-flux hemodialysis range from 200 to 250 mL/min. Higher post-thrombolytic values (at least 300 mL/min) recognized by some authors as optimal blood flow (mostly for high-flux hemodialysis) may lead to an excessive amount of medical interventions, including thrombolytic procedures and catheter replacements, that do not significantly affect the effectiveness of hemodialysis itself [12].

Searching for predictors we found that the occurrence of DM significantly decreases their maintenance (HR = 0.31). It is a new finding that could be however anticipated, as DM is considered as a significant prothrombotic state [13]. Maintenance of the venous catheter is followed by chronic irritation and repeated injury of the endothelial layer of the vein that predispose for the formation of microthrombi [14]. Prothrombotic state in DM is explained by endothelial injury, hyperfibrinogenaemia and platelet hyperreactivity related to increased glucose levels as well as insulin resistance and hyperinsulinemia [15]. It was shown that even strict glucose control does not normalize activation of the coagulation system in DM patients [16].

We failed to prove cancers and previous episodes of thrombosis to diminish maintenance of tunneled catheters after successful thrombolysis, but this may be a consequence of the study size and small numbers of affected subjects.

The results of our study direct attention to prophylaxis of the recurrent catheter-related thrombosis. However, the limited data precludes formulation of the guidelines.

All included patients, before and after the thrombolysis, received LMWH anticoagulation during HD session, and heparin locks that are routinely used in the participating in our collaborating centers. The limited funding precludes routine using of alteplase containing locks [17]. In addition, our patients, prior to the thrombolysis had received prophylaxis, related to the co-existing diseases: 40% – antiplatelet drugs, 21.2% – vitamin K antagonists and 8.2% – LMWH and were recommended to use either vitamin K antagonists or LMWH between HD sessions.

The latest K/DOQI guidelines from 2006 recognized the prophylactic administration of both warfarin and antiplatelet agents as ineffective [8]. Regardless of that, and unconvincing data coming from systematic reviews [18–21], vitamin K antagonists are still frequently used in Poland (both acenocoumarol and warfarin) in the secondary prevention of catheter-related thrombosis. Also some authors, for example Zellweger et al. [22] recommend to restrict its use to the patients with the increased risk of thrombosis. Results of our study suggest that patients with diabetes should be included in this group; however, the evidence is missing.

The therapy should be at least safe for the patient. Therefore, an appropriate tailoring of vitamin K antagonists dose is the key point (to keep INR within the range 1.5–2.0), to prevent the occurrence of bleeding, especially fatal bleeding and hemorrhagic stroke [21].

The use of LMWH in the secondary prevention of catheter-related thrombosis in HD patients on the daily schedule is not supported by evidence based medicine and more expensive than the use of vitamin K antagonists. This shows how urgently needed are randomized studies comparing the efficacy and safety of vitamin K antagonists and LMWH in the prevention of recurrent catheter-related thrombosis.

In our opinion, diabetic patients should be considered as high-risk patients for recurrent catheter-related thrombosis. Therefore, the use of catheter should be restricted to patients with contraindications to create the arterio-venous fistula or patients with multiple failures of creating a workable one. In patients that have to stay on catheter, antithrombotic prophylactics is required. However, there is no data that could support any statement concerning its choice in this group of patients. We can only suggest a conscientious monitoring of vitamin K antagonists therapy to keep INR in the therapeutic range and the use of alteplase [17] as locking solution, if this is not a standard treatment in the center. Perhaps the addition of urokinase to the lock solution may have similar effect, that is, currently tested in the double blind randomized controlled trial [23].

Our study has some limitations. First of all, we did not screen the patients for thrombophilia, that prevalence in Poland is estimated at about 8% in the general population [24]. It may be expected that the prevalence could be greater in our study group. On the other hand, it should be stressed that none of our patients was diagnosed with thrombophilia based on the history of prior thrombosis episodes not related to the catheter insertion.

## Conclusions

The occurrence of DM significantly increase the risk of recurrent catheter-related thrombosis and in consequence decrease one-year catheter survival.
